# RV lead placement – A forgotten cause of right heart failure

**DOI:** 10.1016/j.amsu.2021.102461

**Published:** 2021-06-07

**Authors:** Muhammad Arslan Cheema, Talal Almas, Waqas Ullah, Donald Haas

**Affiliations:** a Thomas Jefferson University, PA, USA; b RCSI University of Medicine and Health Sciences, Dublin, Ireland; c Abington Jefferson Health, PA, USA

**Keywords:** RV lead placement, Right heart failure

## Abstract

**Introduction:**

Cardiac implantable electronic devices (CIEDs) have opened new doors, improving the quality, and increasing the duration of life by providing support of heart rate, atrioventricular and interventricular synchrony, thereby preventing sudden cardiac death. Nevertheless, these devices can pose some risks to the patients, including pacemaker-mediated cardiomyopathy and endocarditis.

**Case presentation:**

We elucidate the case of a patient who had severe Tricuspid Regurgitation as a result of single chamber Implantable Cardioverter Defibrillator (ICD) placement which led to right heart failure (RHF). His chief complaints were generalized fatigability and difficulty climbing steps at home. He also had orthopnea but denies paroxysmal nocturnal dyspnea. Despite using home diuretic regimen (Torsemide 40 gm daily), his continued to increase. He did not respond well to intravenous diuretics that time so decision was made to start Aquapheresis to which he responded very well

**Discussion:**

TV dysfunction associated with CIED leads can be investigated and diagnosed using different techniques. These pillars of diagnostic tests include two-dimensional (2D), 3D, and Doppler echocardiography. Presence of holosystolic hepatic vein flow reversal is key in diagnosing severe TR, whereas normal antegrade systolic flow excludes the possibility of moderate and severe TR.

**Conclusion:**

CIED leads causing tricuspid valve impairment has become increasingly recognized over the recent times; however, the evidence underlying this trend has been derived primarily from retrospective analyses. In order to circumvent these issues, leadless pacemakers and subcutaneous ICD devices should be considered.

## Highlights

•CIED leads causing TV dysfunction has gained increasing recognition recently but all evidence supporting this premise emanates from retrospective analyses.
•When clinical, hemodynamic, and echocardiographic assessment provides compelling evidence of lead related severe TR, corrective intervention should be provided in a timely fashion, before the onset of severe annular and chamber dilation and severe RV dysfunction because, by that time, the lead itself will no longer be the problem, and the extant problem may not be as amenable to corrective intervention.
•Leadless pacemakers and subcutaneous ICD devices likely to be associated with a reduction in lead-related cardiac dysfunction.

## 1 Introduction

Cardiac implantable electronic devices (CIEDs) have opened new doors, improving the quality and increasing the duration of life by providing support of heart rate, atrioventricular and interventricular synchrony, thereby preventing sudden cardiac death [1]. These devices continuously monitor cardiac rhythm and if these devices encounter VT/VF, a shock is promptly delivered to terminate the episode. These devices can pose some risks to the patients like pacemaker mediated cardiomyopathy and endocarditis. We elucidate the case of a patient who had severe Tricuspid Regurgitation as a result of single chamber Implantable Cardioverter Defibrillator (ICD) placement which led to right heart failure (RHF).

## 2 Case presentation

**Fig. 1:**
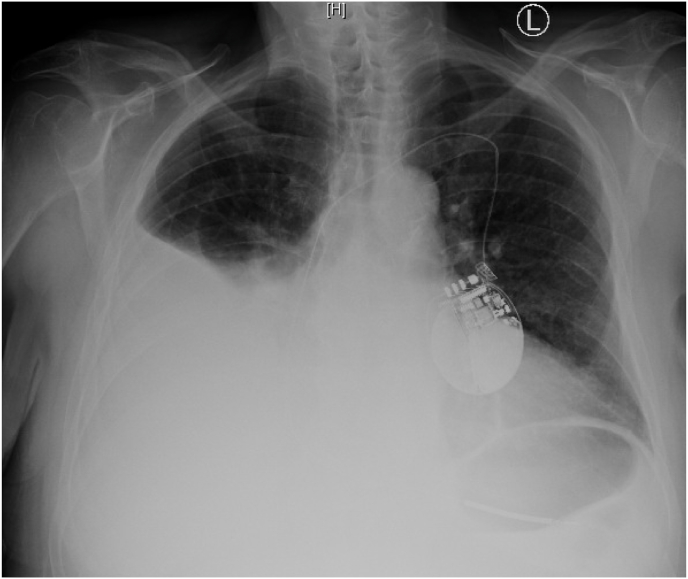
Chest X-ray divulging a large right-sided pleural effusion.

**Fig. 2:**
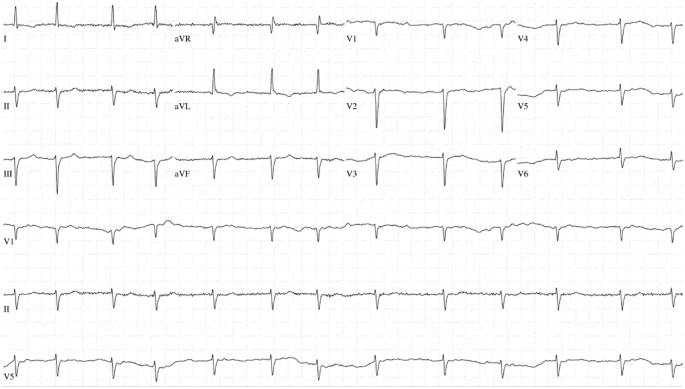
EKG demonstrating a classical atrial fibrillation pattern with left axis deviation.

A 79 year old man with was referred to the emergency room by his primary care physician for worsening shortness of breath going on for 4 weeks. He had previous medical history significant for non-ischemic cardiomyopathy (Ejection Fraction 35%) status post single chamber ICD placement 3 years ago, permanent atrial fibrillation not on anticoagulation due to history of intracranial bleed, hypertension and type 2 Diabetes. His main complaints were generalized fatigability and difficulty climbing steps at home. He also had orthopnea but denies paroxysmal nocturnal dyspnea. Inspite of using home diuretic regimen (Torsemide 40 gm daily), his weight kept on increasing. He was compliant with salt and fluid restriction. He was found to be almost 40 pounds overweight from his baseline. He had jugular venous distension and significant lower extremity edema on examination. His blood work was significant for normal renal function, hepatic function and cardiac BNP was 1109 pg/ml. Chest Xray (Fig. 1) showed large right sided pleural effusion which was tapped and found to be transudative. His EKG (Fig. 2) showed atrial fibrillation and left axis deviation.

**Fig. 3:**
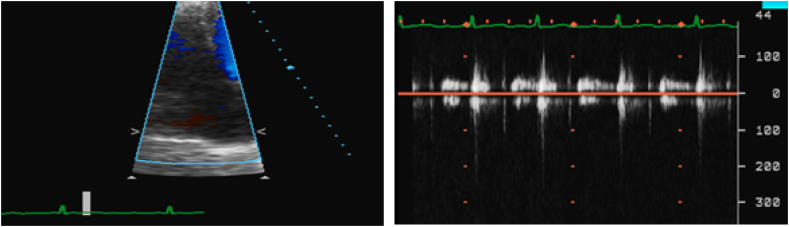
Color & spectral Doppler showing minimal TR. (For interpretation of the references to colour in this figure legend, the reader is referred to the Web version of this article.)

**Fig. 4:**
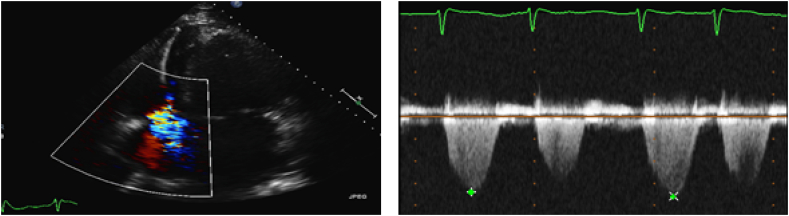
Color & spectral Doppler showing moderate to severe TR. (For interpretation of the references to colour in this figure legend, the reader is referred to the Web version of this article.)

Transthoracic echocardiogram showed 50–55% left ventricular ejection fraction, mildly reduced right ventricular systolic dysfunction and moderate tricuspid regurgitation resulting in moderate pulmonary hypertension. Echocardiogram a year before ICD placement showed trivial TR (Fig. 3, Fig. 4). Right heart catheterization (RHC) showed severely elevated right sided pressures with normal pulmonary vascular resistance. He responded well to intravenous diuretics. His ICD interrogation revealed 22% RV pacing. His heart rate remained in 70–80 beats/minute. He was discharged from the hospital in stable condition. After few months, he was admitted again in the hospital with refractory volume overload (30 pounds above his baseline weight). Again, RHC showed elevated filling pressures. He did not respond well to intravenous diuretics that time so decision was made to start Aquapheresis to which he responded very well. His transthoracic echocardiogram showed further worsening of TR and severely enlarged right atrium/right ventricle. His ejection fraction was found to be preserved.

The patient was followed in the clinic. He was followed up monthly for 1st two months and then advised to follow up every 6 months. Device was interrogated at every visit and no inappropriate therapy was found to be delivered.

## 3 Discussion

**Fig. 5:**
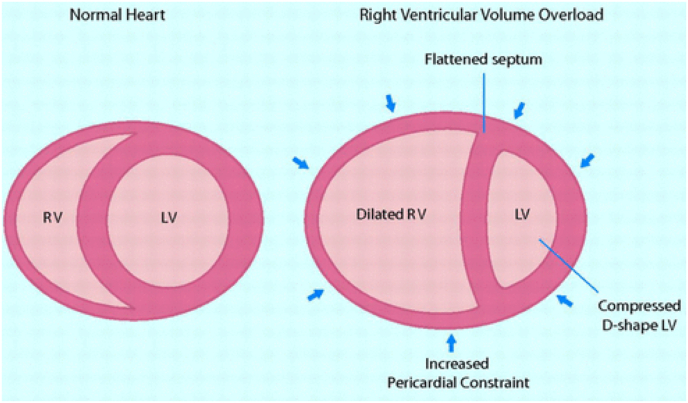
Ventricular interdependence in chronic right heart failure.

Right heart failure (RHF) is a clinical syndrome characterised by symptoms and signs, that arise as a result of dysfunctioning of right heart structures including the right ventricle and the tricuspid valve, leading to decreased ability of the right heart to supply blood to the lungs at normal central venous pressures [2]. Right heart failure can be an acute or chronic process and its aetiology comprises acquired or congenital forms of cardiovascular disease (Fig. 5).

Acute right heart failure can occur due to RV infarction, myocarditis or from pulmonary embolism while the causes of chronic RHF are listed in Table 1.

**Table 1:** A tabulation of the established causes of right heart failure.

Volume Overload	Pressure Overload
Tricuspid Regurgitation	Left sided heart failure
Pulmonary Regurgitation	Pulmonary hypertension
Transposition of Great Vessels	

Normal pulmonary circulation is a low resistance, high-compliance system that accommodates a large volume of blood flow with minimal increase in pressures under normal conditions. Right ventricle normally ejects blood at very low pressure, as compared with the much thicker-walled LV. Thus, the RV is generally far more afterload sensitive than the LV. For this reason, acute increases in pulmonary artery (PA) pressure, such as that caused by acute pulmonary embolism, may precipitate cardiogenic shock because the RV cannot generate sufficient pressure to maintain pulmonary perfusion. With chronic, sustained increases in PA pressure there are hypertrophic adaptations in the RV that allow for maintenance of forward flow despite the increase in RV afterload. Overtime, this adaptive hypertrophy progresses and becomes maladaptive, leading to RV dilation and progressive myocardial dysfunction. With increasing RV volumes, there is dilation of the tricuspid valve apparatus, precipitating functional tricuspid regurgitation. This further increases the right atrium volume load and RV, promoting further dilation and increasing severity of tricuspid regurgitation as part of a ferocious chain of events. Atrial fibrillation often develops in the setting of progressive atrial enlargement, but the relationship is bidirectional, as atrial fibrillation also leads to worsening RV dysfunction.

Impaired RV systolic function, increasing tricuspid valve regurgitation, and impaired LV filling reduce forward stroke volume and cardiac output. This leads to neurohormonal activation that promotes renal sodium and water retention. The consequent volume overload and myocardial dysfunction cause marked elevation in central venous pressure. This leads to an increase in tissue hydrostatic pressures in and decreases lymph flow. Systemic venous hypertension in the body results in gut and lower extremity edema, ascites, and liver dysfunction from congestive hepatopathy that may progress to cirrhosis. Increases in central venous pressure also increase renal vein pressure and play a key role in promoting cardiorenal syndrome.

Most patients with RHF typically have history of known left-sided HF, pulmonary diseases like COPD or obstructive sleep apnea but some patients present with symptoms and signs of RHF (unexplained ascites or dyspnea) without these obvious etiologies. Currently, the universal requirement, involving the use of an endocardial lead for pacing or defibrillation, or both, in the right ventricle has resulted in the identification of various unfavourable outcomes in the context of tricuspid valve (TV) structure and function.

**Fig. 6:**
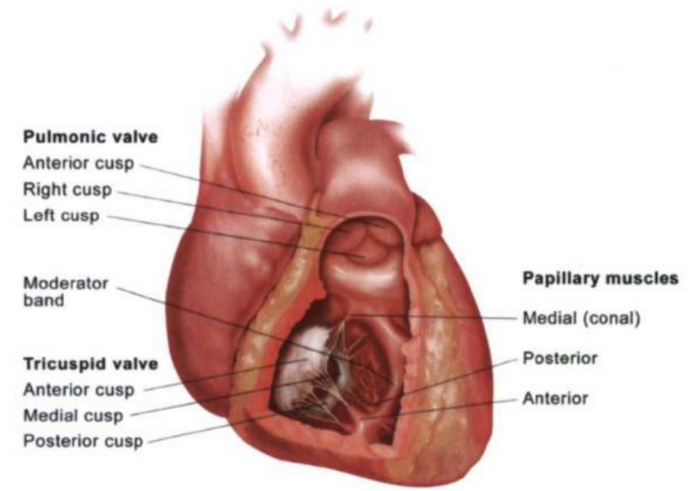
A delineation of the anatomy of the tricuspid valve.

The tricuspid valve apparatus is a complex structure consisting of four components: the leaflets (anterior, posterior, and septal), the non-planar elliptical annulus, two papillary muscles (anterior and posterior), and the chordal attachments Fig. 6. Tricuspid regurgitation (TR) shows signs of worsening due to the effects of prolonged volume overload, including dilatation of chamber and annulus, tricuspid leaflet tethering and reduced mobility, and incomplete coaptation. Therefore, in the presence of left-sided cardiac dysfunction which makes the patient susceptible to TR, even a slight increase in TR associated with the presence of a cardiovascular implantable electronic device (CIED) lead can, with the passage of time, cause severe TR and RH. Our patient had minimal TR prior to placement of right sided defibrillator lead and it got worse after that and resulted in right ventricular dysfunction.

As the severity of TR increases, the survival rates plummet, regardless of ejection fraction or pulmonary artery pressure. The prognosis also gets poorer with increasing severity, with age, right ventricular size, biventricular systolic function, and dilation of the inferior vena cava bearing little effect of the prognosis [3].

Tricuspid valve leaflets or sub-valvular structures can be compromised/damaged during lead implantation or manipulation, in a myriad of forms, and it may not be picked by follow up routine imaging. These forms of structural damage include: leaflet perforation, avulsion which may occur during extraction of leads, laceration and transection of chordal structures or papillary muscles. After implantation of a CIED, mechanical interference with TV leaflet movement and coaptation can cause tricuspid regurgitation. A lead traversing the TV can thwart leaflet coaptation in two ways: direct contact with the leaflets or entwining with chordae tendineae [[4], [5], [6]]. TV dysfunction associated with CIED leads can be investigated and diagnosed using different techniques. These pillars of diagnostic tests include two-dimensional (2D), 3D, and Doppler echocardiography. Presence of holosystolic hepatic vein flow reversal is key in diagnosing severe TR, whereas normal antegrade systolic flow excludes the possibility of moderate and severe TR [7].

Currently, many studies are being conducted to compare transvenous versus subcutaneous ICDs [[8], [9], [10]]. The PRAETORIAN trial is a randomized, controlled noninferiority trial which compared two types of ICDs. At 48 months, the estimated cumulative incidence of the primary end point (composite of device-related complications and inappropriate shocks) was 15.1% in the subcutaneous ICD group and 15.7% in the transvenous ICD group (hazard ratio, 0.99; 95% confidence interval, 0.71 to 1.39; P = 0.01 for noninferiority). There was no significant difference between the two groups regarding the secondary end point of death from any cause. The trial showed that both systems were quite effective at terminating malignant arrhythmias. The trial showed that both systems were quite effective at terminating malignant arrhythmias. However, complications, including lead malfunction and infection, were more common with the transvenous ICD [11,12], whereas inappropriate shocks were more common with the subcutaneous ICD. The risk of inappropriate shock can be mitigated by selecting the appropriate vector in case of subcutaneous ICDs [[13], [14], [15]].

Recently, there has been another study done which favor the use of the subcutaneous ICD for patients with inherited arrhythmia syndromes and genetic cardiomyopathies who do not need anti-bradycardia pacing [15]. A meta-analysis Roberto Rodorf et al. demonstrated that in patients with an indication for ICD without the need for pacing, transvenous ICD and subcutaneous-ICD are overall comparable in terms of the composite of clinically relevant device-related complications and inappropriate shock [15].

This case report was drafted in accordance with the SCARE guidelines [16].

## 4 Conclusions

CIED leads causing tricuspid valve impairment has become increasingly recognized over the recent times; however, the evidence underlying this trend has been derived primarily from retrospective analyses. In the backdrop of clinical and echocardiographic assessment insinuating a diagnosis of the pathology, timely treatment should be carried out in order to avoid cardiovascular ramifications. In order to circumvent these issues, leadless pacemakers and subcutaneous ICD devices should be considered.

## Disclosures

None

## Provenance and peer review

Not commissioned, externally peer-reviewed

## Ethical approval

Obtained.

## Sources of funding

N/A.

## Author contribution

MAC and TA: drafted the initial version of the manuscript and conducted the literature search. WU and DF: diagnosed the case, proofread the final draft, and revised it critically

## Consent

Written informed consent was obtained from the patient for publication of this case report and accompanying images. A copy of the written consent is available for review by the Editor-in-Chief of this journal on request.

## Registration of Research Studies

Name of the registry:

Unique Identifying number or registration ID:

Hyperlink to your specific registration (must be publicly accessible and will be checked).

## Guarantor

Waqas Ullah

## Declaration of competing interest

None
