# Supplementary material for: RV lead placement – A forgotten cause of right heart failure
Source: Ann Med Surg (Lond). 2021 Jun 7;67:102461. doi: 10.1016/j.amsu.2021.102461 (PMC8203726; doi:10.1016/j.amsu.2021.102461)
Supplement: Multimedia component 1 [file mmc1.pdf]

CARE case report guidelines

CARE Checklist of information to include when writing a case report

(cc) BY-NC-ND

Topic	Item	Checklist item description	Reported on Line
**Title**	**1**	The diagnosis or intervention of primary focus followed by the words "case report"	Intro- 3
**Key Words**	**2**	2 to 5 key words that identify diagnoses or interventions in this case report, including "case report"	
**Abstract (no references)**	**3a**	Introduction: What is unique about this case and what does it add to the scientific literature?	
	**3b**	Main symptoms and/or important clinical findings	
	**3c**	The main diagnoses, therapeutic interventions, and outcomes	
	**3d**	Conclusion—What is the main "take-away" lesson(s) from this case?	Case presentation-10
**Introduction**	**4**	One or two paragraphs summarizing why this case is unique (**may include references**)	
**Patient Information**	**5a**	De-identified patient specific information.	
	**5b**	Primary concerns and symptoms of the patient.	
	**5c**	Medical, family, and psycho-social history including relevant genetic information	
	**5d**	Relevant past interventions with outcomes	
**Clinical Findings**	**6**	Describe significant physical examination (PE) and important clinical findings.	
**Timeline**	**7**	Historical and current information from this episode of care organized as a timeline	
**Diagnostic Assessment**	**8a**	Diagnostic testing (such as PE, laboratory testing, imaging, surveys).	
	**8b**	Diagnostic challenges (such as access to testing, financial, or cultural)	
	**8c**	Diagnosis (including other diagnoses considered)	
	**8d**	Prognosis (such as staging in oncology) where applicable	Case presentation-10
**Therapeutic Intervention**	**9a**	Types of therapeutic intervention (such as pharmacologic, surgical, preventive, self-care)	
	**9b**	Administration of therapeutic intervention (such as dosage, strength, duration)	
	**9c**	Changes in therapeutic intervention (with rationale)	
**Follow-up and Outcomes**	**10a**	Clinician and patient-assessed outcomes (if available)	
	**10b**	Important follow-up diagnostic and other test results	
	**10c**	Intervention adherence and tolerability (How was this assessed?)	
	**10d**	Adverse and unanticipated events	Discussion-20
**Discussion**	**11a**	A scientific discussion of the strengths AND limitations associated with this case report	
	**11b**	Discussion of the relevant medical literature **with references**.	
	**11c**	The scientific rationale for any conclusions (including assessment of possible causes)	
	**11d**	The primary "take-away" lessons of this case report (without references) in a one paragraph conclusion	
**Patient Perspective**	**12**	The patient should share their perspective in one to two paragraphs on the treatment(s) they received	
**Informed Consent**	**13**	Did the patient give informed consent? Please provide if requested	**Yes** ☐ **No** ☐ Yes
